# Innovative and Conventional Valorizations of Grape Seeds from Winery By-Products as Sustainable Source of Lipophilic Antioxidants

**DOI:** 10.3390/antiox9070568

**Published:** 2020-07-01

**Authors:** Ivana Dimić, Nemanja Teslić, Predrag Putnik, Danijela Bursać Kovačević, Zoran Zeković, Branislav Šojić, Živan Mrkonjić, Dušica Čolović, Domenico Montesano, Branimir Pavlić

**Affiliations:** 1 Faculty of Technology, University of Novi Sad, Blvd. cara Lazara 1, 21000 Novi Sad, Serbia; ivana.dimic@live.com (I.D.); zzekovic@tf.uns.ac.rs (Z.Z.); sojic@tf.uns.ac.rs (B.Š.); zivan_mrkonjic@hotmail.com (Ž.M.); 2 Institute of Food Technology, University of Novi Sad, Blvd. cara Lazara 1, 21000 Novi Sad, Serbia; nemanja.teslic@fins.uns.ac.rs (N.T.); dusica.colovic@fins.uns.ac.rs (D.Č.); 3 Faculty of Food Technology and Biotechnology, University of Zagreb, Pierottijeva 6, 10000 Zagreb, Croatia; pputnik@alumni.uconn.edu (P.P.); dbursac@pbf.hr (D.B.K.); 4 Section of Food Science and Nutrition, Department of Pharmaceutical Sciences, University of Perugia, Via San Costanzo 1, 06126 Perugia, Italy

**Keywords:** grape seed oil fatty acid, novel extraction, tocopherol, antioxidant activity, supercritical fluid, microwave assisted, ultrasound assisted, Soxhlet

## Abstract

The aim of this study was to valorize the oil recovery from red and white grape seeds (*Vitis vinifera* L.) that remains as by-product after the winemaking process. Oils were extracted by modern techniques, ultrasound assisted (UAE), microwave assisted (MAE) and supercritical fluid extraction (SFE), and compared to the Soxhlet extraction (SE). Firstly, SFE was optimized at different operating conditions: pressure (250–350 bar), temperature (40–60 °C), CO_2_ flow rate (0.2, 0.3 and 0.4 kg h^−1^), and particle size (315–800 µm and >800 µm). The highest extraction yields were achieved by SFE at the optimal conditions: 350 bar, 60 °C, 0.4 kg h^−1^. Afterwards, SFE was compared to SE, UAE and MAE with respect to oil extraction yields, and analyzed for fatty acid composition and antioxidant capacity. Considering the general classification of fatty acids, it was found that samples had high content of polyunsaturated fatty acids, regardless of extraction technology. Tocopherol content was significantly influenced by all extraction methods, whereas UAE and MAE resulted in extracts richer with lipophilic antioxidants. In conclusion, modern extractions that are suited for industrial applications had better performance as compared to SE, as judging by the oil yield and quality.

## 1. Introduction

Grape processing generates large quantities of important agricultural and industrial wastes/by-products with potential to be reused for various purposes. It has been estimated that more than 0.3 kg of solid by-products is allocated per kg of mashed grape fruit during the processing [1]. The waste streams of wine production contain organic waste, greenhouse gases (CO_2_, vaporous compounds etc.) and non-organic waste (diatomaceous earth, bentonite clay, perlite). In particular, organic waste, as grape pomace with seeds, pulp and skins, grape stems and leaves, represents about two thirds of entire solid waste [2,3]. The handling and disposal of this great amount of waste/by-products is a large environmental problem [3,4]. Currently, new processes for the controlled waste removal are being searched, targeting the conversion of the waste material and its incorporation into new bio-products with added value.

Seeds are the major material of the industrial processing of grape berry (e.g., found in pomace) and constitute about 7–20% of the weight of grapes processed [5]. Although grape seeds are mutually removed with the skins and vascular fruit tissues from the pomace, they can be easily separated through technological separation and sieving [6]. Although grape peels and stems do not have an economic background for industrial utilization, seeds are rich in bioactive antioxidants, and can be raw materials for the development of new foods, as natural extracts, pharmaceutical products [7,8] and cosmetics. Therefore, the production of grape seed oil contributes to the advance of waste management that could increase the financial income of the primary industrial process and sustainability [4].

The oil content of grape seeds from the literature was reported in the range of 13–15% depending on the variety and maturity of grapes [9]. The interest in grape seed oil as a functional food product has increased, particularly because of its high levels of lipophilic ingredients, such as vitamin E, unsaturated fatty acids (UFAs), and phytosterols [10] that possess greater antioxidant activity than hydrophilic ingredients [9]. Namely, grape seed oil was identified as a rich source of tocopherols, tocotrienols and unsaturated fatty acids, especially in polyunsaturated fatty acids (PUFAs), whereas linoleic acid (C18:2) was found as predominant (49.0–78.2% of total PUFAs) [4]. γ-tocotrienol was evidenced as the most abundant tocotrienol, followed by α-tocotrienol, while δ-tocotrienol was found in lower amounts [11]. Tocopherols from seed oils are α-, β-, γ-, and δ-tocopherol, with α-tocopherol as one of the most potent intracellular fat-soluble antioxidants due to its activity in inhibiting the peroxidation of polyunsaturated fatty acids in biological membranes [4]. A mixture of α-tocopherol and α- and γ-tocotrienol purified from grape seeds was more effective than other lipophilic grape seed fractions in neutralizing free and lipid peroxy radicals and in chelating prooxidant metals [12]. Therefore, grape seed oil extract can be considered a valuable source of natural liposoluble antioxidants, having potential health benefits [13,14].

On the industrial level, grape seed oils are mainly produced by traditional oil extraction methods, such as cold-press and solvent extraction [15,16]. Traditional processing often leads to a higher solvent consumption, longer extraction times, lower yields and poorer extraction quality [17]. As compared to Soxhlet extraction, cold pressing has potential for higher yields of fatty acids and tocopherols, without the assistance of heat and chemical treatment. Thus, cold-pressed oils are interesting raw materials for natural and safe food products favored by manufacturers and consumers [18].

Commonly, Soxhlet extraction employs *n*-hexane as solvent for grape oils, which is not selective and simultaneously removes non-volatile pigments and waxes. Consequently, the obtained extracts are dark, viscous and contaminated with the traces of toxic solvent [19]. Many contemporary extraction technologies avoid the negative impacts of thermal degradation and meet the criteria for the “green“ extraction processes [20]. Aside from that, they offer energy savings, either minimize or avoid the use of organic solvents, shorten the processing time, reduce the temperature, enhance the mass transfer process, and increase the extraction yield with high quality extracts [21]. Extractions aided by ultrasound, microwave or high-pressure processing are being explored as alternative technologies for intensification of the extracted antioxidants from grape seeds.

Ultrasound-assisted extraction (UAE) is based on the acoustic cavitation phenomenon. The period of negative pressure during the ultrasound treatment causes bubbles, hence the origins of increased pressure and temperature with their subsequent collapse. When this happens, the resulting ”shock waves“ break the cellular walls and facilitate solvent penetration into plant materials which enhances extraction yields [22]. Microwave-assisted extraction (MAE) uses microwaves that are nonionizing, electromagnetic waves with the frequency between 300 MHz and 300 GHz. Here, electromagnetic waves are transformed to thermal energy, which induces heating of the matrix on inside and outside without thermal gradient. If a sufficient amount of thermal energy is generated, this local heating damages cell wall of plant matrix and causes leakage of target compounds into extraction medium. In the literature, MAE was useful for extraction of biologically active substances with antioxidant properties from grape seeds [23,24]. Supercritical fluid extraction (SFE) represents another excellent alternative to conventional extraction with the potential to achieve comparable yields. Additionally, grape seed oils recovered by SFE are characterized by higher product quality that is similar to mechanical pressing [25]. Supercritical fluids, especially CO_2_, have the gas-like properties, e.g., viscosity and diffusivity, and liquid-like properties, e.g., density and solvation power [26]. CO_2_ is a green, low-cost, non-toxic and non-flammable solvent with critical pressure of 73 bar and temperature of 31 °C. It can be re-used in processing, hence its ability to reduce total energy costs in industry [27]. In addition, the residues of the solvent do not remain in the final product, because supercritical CO_2_ can be completely eliminated by pressure reduction [9]. Co-solvents and modifiers (e.g., ethanol, methanol, acetone) may be added to improve the solubility of polar phytochemicals embedded in the cell wall [27]. Moreover, supercritical CO_2_ ensures selectivity in the extraction of certain target compounds by varying operating conditions (e.g., temperature and pressure) [27], while an approximate economic estimate of industrial SFE scale-up from the laboratory is already available from the literature [28].

Since there are no abundant data in the literature for comparison of alternative vs. conventional extractions of grape seed oils with potential for industrial applications, the aim of this study was to compare Soxhlet against UAE, MAE and SFE concerning efficiency for obtaining high-quality extracts. In this work, we postulated that SFE is an important green alternative to organic solvent extraction for recovery of lipophilic antioxidants from winery waste streams by considering extraction parameters, in-depth chemical profiling, functional qualities and bioactivity of samples. Samples were compared in terms of total extraction yields, fatty acid profiling, tocopherol content, and antioxidant properties.

## 2. Materials and Methods

### 2.1. Plant Material

The industrial by-products of a various white and red grapes (*Vitis vinifera* L.) containing separated seeds were received from Kovačević Winery D.O.O. (Irig, Serbia). Red grape seed samples were a mixture of Cabernet Sauvignon, Merlot and Pinot noir with approximate ratio of 65:30:5 (*m*/*m*/*m*), while the white ration of grape varieties in the white seed sample was 60:30:10 (*m*/*m*/*m*) for Chardonnay, Sauvignon blanc and Riesling. The seeds were immediately milled in a domestic blender (Bosch, MMB21P0R/01, Germany) and subjected to extraction of bioactive antioxidants. Mean particle size of the sample was determined by sieving through the vibro-sieve set (CISA Cedaceria Industrial, Spain) with 0.1-, 0.315-, 0.8-, 2- and 4-mm pore size. The mean particle size for the red and white grape seeds was 0.578 and 0.566 mm, respectively. Red grape fractions had 0.315–0.8-mm particle size, and >0.8 mm were used to evaluate the granulation on SFE samples.

### 2.2. Chemicals and Reagents

Helium (>99.9997%) and carbon dioxide (99.9%) were purchased from Messer Technogas A.D., Novi Sad, Serbia. *n*-Hexane was purchased from Merck KgaA, Darmstadt, Germany. Ethanol was purchased from Sani-Hem D.O.O., Novi Bečej, Serbia. Methanol was purchased from Lach-ner Ltd., Neratovice, Chech Republic. Ethyl acetate was purchased from Zdravlje Leskovac, Leskovac, Serbia. 1,1-Diphenyl-2-picrylhydrazyl-hydrate (DPPH), 2,2′-azino-bis(3-ethylbenzothiazoline-6-sulfonic acid) diammonium salt (ABTS) and Trolox (6-hydroxy-2,5,7,8-tetramethylchroman-2-carboxylic acid) were purchased from Sigma Aldrich, St. Louis, MO, United States. Supelco 37 component fatty acid methyl esters (FAMEs) mix, DL-α tocopherol (99.9%), rac-β-tocopherol (99%), γ-tocopherol (97.3%) and δ-tocopherol (95.2%) were purchased from Supelco Inc., Bellefonte, PA, United States.

### 2.3. Extraction Techniques

#### 2.3.1. Soxhlet Extraction (SE)

Grape seeds samples (30.0 g) were extracted using 120 mL of *n*-hexane in Soxhlet apparatus. Extraction was conducted for 6 h with 15 exchanges of the extracts and filtration of the solvent. Extraction solvent was then evaporated under vacuum at 40 °C. Obtained extracts were placed in a glass vials and kept at 4 °C until analysis. Extraction experiments were performed in triplicates and total extraction yield was expressed as mean ± standard deviation.

#### 2.3.2. Ultrasound-Assisted Extraction

Ultrasound-assisted extraction was performed with sonication bath (EUP540A, Euinstruments, Paris, France) on constant frequency 40 kHz. Mass of 30.0 g of grape seeds sampled into a 500-mL glass flask and mixed with 300 mL of *n*-hexane. The condenser was put on glass flask to avoid solvent evaporation. The UAE was assessed using the modified method from the literature [29], with extraction conditions of *T* = 50 °C, *t* = 40 min, and sonication power at 60 W L^−1^. Afterwards, the extracts were filtered and the extraction solvent was evaporated under vacuum at 40 °C. Obtained extracts were placed in glass vials and kept at 4 °C until analysis.

#### 2.3.3. Microwave-Assisted Extraction

Microwave-assisted extraction was performed in experimental setup previously described [30]. Briefly, seeds samples of 10.0 g were mixed with 100 mL of *n*-hexane in glass flask, and placed in microwave extractor with connected condenser through a hole at the top of the casing. Matrix-to-solvent ratios were adjusted according to the cited references and limitations of the experimental setup. Extraction was performed at constant microwave irradiation power (600 W) for 15 min. The obtained extracts were filtered and solvent was removed under vacuum at 40 °C, then placed in glass vials and kept at 4 °C until analysis.

#### 2.3.4. Supercritical Fluid Extraction

Supercritical fluid extraction was done at laboratory scale with high-pressure extraction equipment (HPEP, NOVA-Swiss, Effretikon, Switzerland). The main characteristics of supercritical fluid extractor were described in previous research [31]. Since, preliminary results shown that red varieties seeds contained higher amounts of oils, SFE operating conditions were initially optimized for red grape, and the best SFE conditions were applied to white grape. Red grape samples (100.0 g) were extracted for 4 h at different operating conditions: (i) pressures (250, 300 and 350 bar); (ii) temperatures (40, 50 and 60 °C); (iii) CO_2_ flow rates (0.2, 0.3 and, i 0.4 kg h^−1^); and (iv) particle size fractions (315–800 and >800 µm). A one-factor-at-a-time experimental design was used and sample labels and process conditions listed in Table 1. All extracts were collected in glass vials and stored at 4 °C until analysis.

#### 2.3.5. Extraction Yield

The extraction yield for all applied extractions was calculated according to the following Equation (1):

(1) $$Y\left[\%\right]=\frac{mass\;of\;extracted\;oil}{mass\;of\;wheat\;germ}\times 100$$

### 2.4. Chemical and Antioxidant Characterization of Grape Seeds Oils

#### 2.4.1. Fatty Acid Profiles

Fatty acid methyl esters were prepared from the extracted lipids using a method based on 14% boron trifluoride–methanol solution [32]. Nitrogen was used for drying and removing the solvent from fatty acid methyl esters. Obtained samples were analyzed on GC Agilent 7890A system with FID, automated liquid injection module, equipped with capillary column with silica gel (SP-2560, 100 m × 0.25 mm, I.D., 0.20 µm, Supelco Analytical, Bellefonte, PA, United States). Temperature regime during analysis was set as followed: initial temperature was 140 °C with hold of 5 min, heating up to 240 °C was with 2 °C/ min and hold on 240 °C was 5 min. Helium was used as carrier gas (flow rate = 1.26 mL min^−1^). Fatty acid peaks in samples were identified by comparison with retention times of the standards from Supelco 37 component FAMEs mix and data from internal data library, based on earlier experiments and GC/MS analysis. The results were expressed as a mass of fatty acid or fatty acid group (g) per 100 g of oil.

#### 2.4.2. Functional Quality

The functional quality of grape seed oils was determined by three indices obtained and calculated from fatty acid (FA) profiles. The ratio between hypocholesterolemic and hypercholesterolemic FAs (H/H) was calculated according to Equation (2) [33,34].

(2) $$\frac{H}{H}=\frac{C18:1+C18:2+C18:3}{C14:0+C16:0}$$

Furthermore, the atherogenicity index (AI) and thrombogenicity index (TI) were calculated according to the Equations (3), (4) [33,35].

(3) $$\mathrm{AI}=\frac{C14:0+4\left(C16:0\right)}{\sum MUFA+\sum \omega -3+\sum \omega -6}$$

(4) $$\mathrm{TI}=\frac{C14:0+C16:0+C18:0}{0.5(\sum MUFA)+3\sum \omega -3+0.5\sum \omega -6+\left(\frac{\sum \omega -3}{\sum \omega -6}\right)}$$

where C14:0 is myristic acid, C16:0 is palmitic acid, C18:0 is stearic acid, C18:1 is oleic acid, C18:2 is linoleic acid, C18:3 is α-linolenic acid. ∑MUFA is a sum of monounsaturated FAs, Σω-3 sum of the polyunsaturated ω-3 FAs and Σω-6 is sum of the polyunsaturated fatty ω-6 acids.

#### 2.4.3. Tocopherols

Tocopherol content was determined by high-pressure liquid chromatography (HPLC), according to the modified method from the literature [36]. Samples were diluted in *n*-hexane and filtered through an RC 0.45-μm syringe filter (Agilent Technologies Inc., Böblingen, Germany). HPLC system (Agilent liquid chromatography series 1260) was equipped with quaternary pump, autosampler and fluorescence detector (Agilent Technologies Inc., Böblingen, Germany). Separation of tocopherols was carried out with normal-phase Luna^®^ 5 μm Silica (2) 100ALC Column (250 × 4, 6 mm) analytical column (Phenomenex, Torrance, CA, United States) and 10 min isocratic analysis run with tetrahydrofurane/*n*-hexane mixture (4:96, *v*/*v*) as mobile phase with flow rate 1.3 mL min^−1^. The column was thermostatted at 35 °C with an injection volume of 5 μL. Fluorescence detector was set at 290-nm excitation wavelength and 330-nm emission wavelength. For each tocopherol, standard stock solutions were prepared and treated as samples in the following fractions: α-tocopherol (0.5–50.0 ppm), β-tocopherol (0.5–50.0 ppm), γ-tocopherol (0.2–25.0 ppm), and δ-tocopherol (0.5–25 ppm). The external calibration curves were made and used for identification and quantification. The results were expressed as mg of tocopherol per g of grape seed oil (mg g^−1^ oil).

### 2.5. Determination of In Vitro Antioxidant Capacity

The in vitro antioxidant capacity was evaluated by two methods: DPPH (2,2-diphenyl-1-picrylhydrazyl) and ABTS (2,2′-azino-bis-(-3-ethylbenzothiazoline-6-sulfonic acid) diammonium salt).

#### 2.5.1. DPPH Assay

The capacity of samples towards scavenging of 1,1-diphenyl-2-picrylhydrazyl-hydrate (DPPH) radicals was measured by published method [37] with slight modifications for lipid samples [38]. Briefly, methanol solution of DPPH reagent (65 µM) was freshly prepared and adjusted with methanol to reach 0.70 (± 0.02) absorbance. Volumes of 0.1 mL of samples were diluted in ethyl acetate, and mixed with 2.9 mL of DPPH reagent in a glass tubes and incubated in dark for 60 min. Blanks were prepared by mixing 0.1 mL of ethyl acetate and 2.9 mL of DPPH reagent. Free radical scavenging measurements were performed in triplicates at 517-nm wavelength by UV/Vis spectrophotometer (6300 Spectrophotometer, Jenway, Staffordshire, UK). Freshly prepared Trolox methanolic solutions (1.33−26.64 µM) were used for the calibration curves. The obtained results were expressed as µM of Trolox equivalents per g of grape seed oil (µM Trolox/g).

#### 2.5.2. ABTS Assay

The ability of samples towards scavenging of ABTS radicals was measured by modified method from the literature [39]. Briefly, ABTS stock solution was freshly prepared from the mixture (1:1, *v*/*v*) of 2.45-mM potassium persulphate aqueous solution and 7-mM ABTS aqueous solution, then left to sit in a dark area at room temperature for the next 16 h. Stock solution was diluted using 300-mM acetate buffer (pH = 3.6) to reach 0.70 (± 0.02) absorbance. A volume of 0.1 mL of a sample (diluted in ethyl acetate) and ABTS reagent (2.9 mL) were mixed and incubated in a dark for 5 h. The blank was obtained by mixing 0.1 mL of ethyl acetate and 2.9 mL of ABTS reagent. Absorbance was measured at 734 nm in triplicates by UV/Vis spectrophotometer (6300 Spectrophotometer, Jenway, Staffordshire, UK). Freshly prepared Trolox ethanolic solutions (0.8–26.6 µM) were used for the calibration curve. The results were expressed as µM of Trolox equivalents per g of grape seed oil (µM Trolox/g).

### 2.6. Statistical Analysis

All experiments were performed in triplicates and results were presented as mean value ± standard deviation (SD), while significant levels were defined at *p* ≤ 0.05 using Tukey’s test. Statistical analysis was carried out using Statistica 10.0 (StatSoft Inc., Tulsa, OK, USA).

## 3. Results

### 3.1. Influence of SFE Operating Parameters on Total Extraction Yield

**Figure 1:**
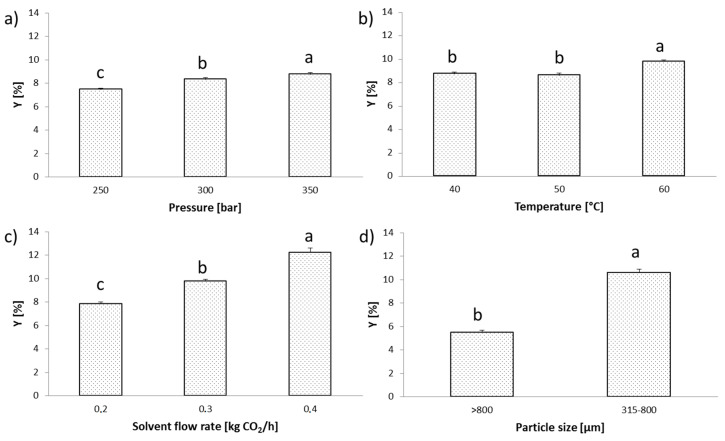
Influence of supercritical fluid extraction (SFE) extraction parameters on total extraction yield of grape seed oil: (**a**) Influence of pressure at fixed temperature (40 °C) and CO_2_ flow rate (0.3 kg/h), (**b**) Influence of temperature at fixed pressure (350 bar) and CO_2_ flow rate (0.3 kg/h), (**c**) Influence of CO_2_ flow rate at fixed pressure (350 bar) and temperature (60 °C), and (**d**) Influence of particle size at 350 bar, 60 °C and 0.4 kg CO_2_/h. * Different letters indicate significant difference (*p* ≤ 0.05) between total extraction yields of grape seed oil with respect to sources of variation.

Preliminary research identified red grape seeds as richer in oil, so it was selected as a gauge for SFE optimization. SFE was conducted upon various extraction parameters of pressure with respect to the total extraction yield of red grape seed oil (Figure 1). Experimentally obtained SFE yields ranged from 7.46 to 12.23%. The highest yield was achieved at following operating conditions: pressure of 350 bar, temperature of 60 °C and CO_2_ flow rate of 0.4 kg h^−1^; therefore, these values were selected as the optimal parameters for further experimentations. 

By increasing pressure from 250 to 350 bar, with other constant parameters (*T* = 40 °C and CO_2_ flow rate of 0.3 kg h^−1^), the extraction yield raised from 7.46 to 8.73% (Figure 1a). An increase in pressure at isothermal conditions caused the density of the supercritical CO_2_ to increase as well, which improved its solvating power and dissolution rate, thus improving extraction efficiency [25]. 

The influence of temperature was observed at this constant pressure and CO_2_ flow rate (0.3 kg h^−1^) for different temperature values (40, 50 and 60 °C) (Figure 1b). The highest yield was found at 60 °C (9.80%) and the lowest at 50 °C (8.61%). 

The impact of CO_2_ flow rates on total SFE yield was studied at the constant pressure (350 bar) and temperature (60 °C). Extraction kinetics for CO_2_ flow rates of 0.2, 0.3 and 0.4 kg h^−1^ is depicted in Figure 1c. The highest yield was achieved at the highest applied flow rate (12.23%) and the crucial differences were observed when flow rate of CO_2_ increased.

A further aim in this study was to determine the influence of the particle size on total extraction yields that were previously detected as optimal (i.e., 350 bar, 60 °C, 0.4 kg h^−1^ CO_2_). The influence of particle size on the yield was investigated in two sample fractions obtained by vibro-sifting. The first fraction consisted of red grape samples with particle size between 315 and 800 µm (R315-SFE) and the second one referred to the red grape with particles above 800 µm (R800-SFE). Figure 1d shows that reduced particle size lead to the significant increase in oil yield, which was 10.58% for R315-SFE fraction and 5.49% for R800-SFE fraction. 

### 3.2. Influence of Different Extraction Techniques on Total Extraction Yield

**Figure 2:**
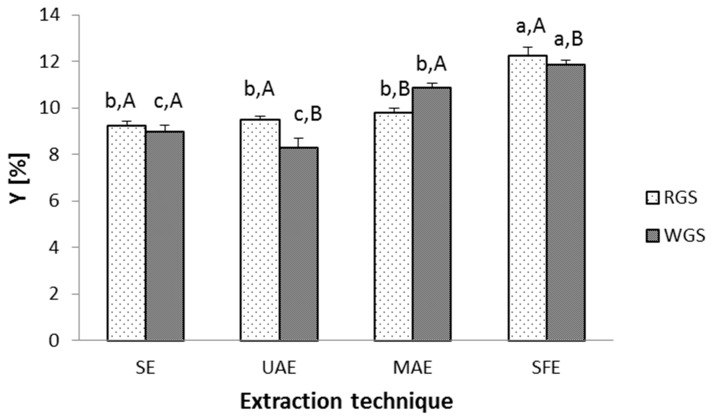
Total extraction yield for grape seed oils recovered by conventional and modern extraction techniques. * Different lowercase letters indicate significant difference (*p* ≤ 0.05) between extraction techniques, while different uppercase letters indicate significant difference between red grape seeds (RGS) and white grape seeds (WGS).

After considering the influence of pressure, temperature, CO_2_ flow rate and particle size, the optimal extraction parameters were selected, and supercritical CO_2_ extraction was performed for red grape seeds (RGS) and white grape seeds (WGS) according to design of an experiment from Table 1. The comparative analysis between conventional (SE) and modern extraction techniques, including UAE, MAE and SFE, was also performed (Figure 2). 

The highest yield was achieved by supercritical CO_2_ extraction at the optimal conditions. SFE yields were 12.23 and 11.86% for red and white grape seeds, respectively. Accordingly, it can be observed that SFE gave the highest total extraction yield as compared to the SE and to all other tested advanced techniques. MAE showed good extraction yield; however, no previous studies were found with confirming suitability of MAE to isolate oils from grape seeds. Therefore, the results from Figure 2 support the use of the MAE for this purpose as well. This is important, as MAE has the advantage over SE due to reducing the extraction time and allowing for lower temperatures that will decrease deterioration of thermolabile oily compounds in the process.

### 3.3. Fatty Acid Profile and Functional Quality

The fatty acid profile of grape seed oils from red and white grape varieties was investigated for all extraction techniques (Table 2). According to the results, it can be seen that samples contained saturated fatty acids (11.28–12.27%), monounsaturated fatty acids (13.53–18.62%), and polyunsaturated fatty acids (69.27–74.88%). Irrespective of extraction type (SE vs. SFE, UAE MAE) or grape variety (red vs. white), eight fatty acids were determined in all samples. Among saturated fatty acids, palmitic acid was predominant one (7.20–7.93%), followed by stearic acid (3.79–4.37%). Monounsaturated oleic acid was present between 13.39–18.47%. Linoleic acid was the most abundant polyunsaturated fatty acid in the investigated samples, accounting for the 68.61–74.15% of all present total fatty acids. Previously, Rodríguez and Ruiz [4] reported that polyunsaturated fatty acids (PUFAs) were predominant in the grape seed samples with 49.0–81.6%, followed by monounsaturated fatty acids (MUFAs) in a range from 13.9–29.1%, and saturated fatty acid (SFAs) that were in a range from 9.6–26.7%. Regarding the fatty acid profiles, palmitic acid (C16:0) dominated in the group of SFAs with 6.7–12.8%, followed by stearic acid (C18:0) that ranged from 2.5% to 15.0%. Concerning the MUFA contents, the oleic acid (C18:1) was found as the major contributor with 0.1–28.9% from the samples. 

The chemical profile of fatty acids could be a useful parameter for the assessment of functional qualities of grape seed oil. The polyunsaturated/saturated fatty acids ratio (PUFA/SFA) is often used to measure indices for frequent cardiovascular disease syndromes (atherogenicity and thrombogenicity), since only three SFAs are hypercholesterolemic [35]. Hence, we calculated the hypo- and hypercholesterolemic fatty acids ratio (H/H), atherogenicity index (AI) and thrombogenicity index (TI) in Table 3.

H/H values ranged from 11.07 to 12.28 for red grape, and from 11.30 to 12.09 for white grape seed oils. The highest H/H in the samples was observed for SE, while for advanced extractions, white grape samples recovered by UAE had the highest H/H value. A higher level of this index is desirable for nutrition, since it expresses the effect of the fatty acids on cholesterol metabolism. For instance, healthy oils as linseed have higher H/H index than grape oils (13.24), sesame and olive oils have lower values [40].

AI values were 0.081–0.090 for red grape seed oil and 0.083–0.088 for white grape samples. TI values were in a range 0.242–0.268 for red grape seed oil and 0.256–0.268 for white grape. The literature reports lower values of AI and TI for linseed oil [40], while sweet cherry, pomegranate, pumpkin [41], sesame and olive oil have higher values [40]. 

### 3.4. Tocopherol Content

Tocopherols, as natural antioxidants, prevent food oxidation processes by preserving oil and fat stability [42]. The tocopherol contents from grape seed oils recovered by SE, UAE, MAE and SFE were determined. The impact of SFE parameters on tocopherol content in grape seed oil was also studied (Table 4). According to obtained results, α-tocopherol was more abundant than γ-tocopherol in all investigated SFE extracts, while β-tocopherol and δ-tocopherol were not even detected. Analyzed tocopherol constituents of the seed oil extracted from 21 grape varieties (Vitis spp.) from Sabir et al. [43] also revealed α-tocopherol as the major constituent form the similar samples.

All of the SFE parameters showed significant effects on the content of both, total and individual tocopherols. Oil sample RGS6-SFE (red grape; *P* = 350 bar; *T* = 40 °C and CO_2_ flow rate 0.2 kg h^−1^) showed the highest total tocopherol content of 9.49 mg 100 g^−1^. Moreover, it can be clearly seen that the content in oil decreased with the increased CO_2_ flow rate. A similar occurrence was observed in the study of Bravi et al. [44], where concentration of α-tocopherol was highest in the first extraction step, when the CO_2_-to-solids mass ratio was 25 g CO_2_ g^−1^ of grape seeds, and further decreased throughout the duration of the process.

One of the aims of the study was to compare different extraction techniques in terms of total and individual tocopherol content (Table 5). By comparing various extractions, it was confirmed that the individual and total tocopherol content was significantly influenced by all the of the extraction methods. In particular, by another non-conventional extraction, i.e., MAE that was also successful in recovering tocopherol-rich samples, where their total content for red and white grapes was 7.96 mg 100 g^−1^ and 2.63 mg 100 g^−1^, respectively. In addition, after calculating the yield of total tocopherols per 100 g of grape seeds, MAE was useful for recovery of grape seed oil with the highest tocopherol yield of 0.778 and 0.286 mg 100 g^−1^ for red and white grape seeds, respectively.

The achieved total tocopherol yield was 0.796 mg 100 g^−1^ for red grape seeds oil sample, i.e., RGS7-SFE. The results of tocopherols yields are shown in Table S1. When compared to other extraction techniques (Table S2), SFE repeatedly displayed highest performance, with tocopherol yields of 0.796 mg 100 g^−1^ for red grape (RGS7-SFE) and 0.286 mg 100 g^−1^ for white grape samples (WGS-SFE). 

### 3.5. In Vitro Antioxidant Capacity

**Figure 3:**
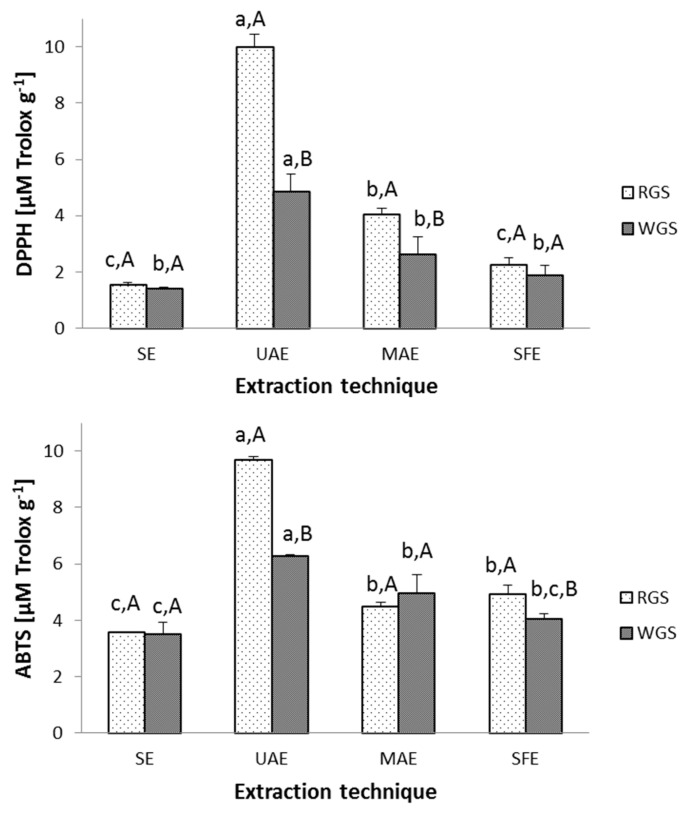
Antioxidant capacity of grape seed oils recovered by conventional and modern extraction techniques (µM Trolox g^−1^). * Different lowercase letters indicate significant difference (*p* ≤ 0.05) between extraction techniques, while different uppercase letters indicate significant difference between RGS and WGS.

The antioxidant capacity of grape seed oils was determined by in vitro DPPH and ABTS assays. The changes in antioxidant capacity influenced by different SFE parameters were given in Table 6. Supercritical CO_2_ extraction proved its efficiency for recovering oil extracts with high antioxidative potential. Samples which were obtained at optimum conditions (RGS7-SFE), had the highest antioxidant capacity as measured by the DPPH assay, while sample RGS1-SFE showed the highest antioxidant capacity with the ABTS assay. Furthermore, differences in the antioxidant capacity of grape seed oils recovered by conventional and non-conventional extractions were shown in Figure 3. The UAE was found to be more efficient for recovery of oils with high antioxidant potential. 

Scavenging activity towards DPPH radicals was between 1.33 and 9.97 µM Trolox g^−1^ for red grape seed oil and between 1.41 and 4.85 µM Trolox g^−1^ for white grape seed oil (Figure 3). For ABTS assay, red grape samples had antioxidant capacity in the range from 3.14 to 9.67 µM Trolox g^−1^, while white grape seed oil ranged from 3.48–6.27 µM Trolox g^−1^ (Figure 3). 

## 4. Discussion

SFE is frequently used as modern technique for the isolation of grape seed oil, therefore it was chosen for optimization and further comparison with Soxhlet extraction and advanced extractions (e.g., UAE and MAE) accounting for yields and lipophilic antioxidant potential. The results of SFE at different pressures were well within the references in the literature, as similar findings were observed by Jokić et al. [39] who performed SFE under experimental conditions of similar pressure (158.58–441.42 bar) and temperature (35.86–64.14 °C) and reached yields from 2.56 to 14.87% for the Cabernet Franc grape variety. In another study, Rombaut et al. [16] investigated the influence of pressures (230–538 bar), temperatures (75–120 °C) and flow rates (5–17 kg h^−1^) and observed total extraction yields from 5.7 to 17.2%. Moreover, Prado et al. [40] observed yield of 13.42% at 350 bar, 40 °C and 0.46 kg CO_2_ h^−1^. The one-factor-at-a-time approach was applied in this work; therefore, after concluding that the highest yield was achieved at 350 bar, that parameter was kept constant for further experimentations. The temperature increase expedited the extraction kinetics by causing a crossover phenomenon. The increased temperature caused a decrease in CO_2_ density resulting in reduced solubility which negatively affected extraction rates [45]. Simultaneously, vapor pressure of the solute increased solubility and positively affected extraction yields. With isobaric increase in temperature, plots of solubility intersected, and these junctures were labeled as “lower and upper crossover points.” At pressures between these two points, solubility decreased with temperature increase, since solvent density overcome the vapor pressure effect. With vapor pressure outside the upper or lower crossover points, its effect become stronger than the density effect, thus the solubility increased with higher temperatures [1].

Passos et al. [19] performed supercritical extraction of Touriga Nacional grape seed oil samples at different pressures and temperatures and concluded that an enhanced extraction rate was achieved with increased pressure and decreased temperature. That was associated with their effects on oil’s solubility and mass transfer coefficients. Coelho et al. [26] observed that higher yields may be achieved at lower pressures and temperatures, as with increased pressure, temperature influence becomes insignificant. Therefore, after conducted experiments, it was concluded that the optimal temperature for SFE was 60 °C, and this temperature was used for further SFE experiments.

A similar influence of solvent flow rate was observed by Duba and Fiori [1], who concluded that increased supercritical CO_2_ flow rates positively affected extraction rates due to external and internal mass transfer. For commercial usages, it was concluded that the solvent flow rates must be optimized in terms of the extraction time and solvent volume, since the increase of CO_2_ flow rates increases specific solvent consumption. Molero Gómez et al. [46] raised flow rates from 0.5 to 2.0 L min^−1^ at the constant conditions (40 °C and 350 bar), and found no significant differences for the yields. The investigated flow rate reached maximum yield of 96% after 3 h of extraction, while lower flow rates took longer time to achieve maximum yields. Finally, the optimal CO_2_ flow rate was defined at constant pressure and temperature at 0.4 kg h^−1^.

Milling the samples facilitated a higher release of oil from the seed cells and shorter diffusion paths in a solid matrix [45]. Molero Gómez et al. [46] have shown that higher extraction yield was obtained with reduced particle size of the samples. As it can be seen from obtained results, the size of the milled grape seeds should be ≥ 350 µm to achieve better efficiency. Total extraction yields may be increased by reducing particle size, which allows higher release of oils from milled particles, due to the widening of the surface area [47].

Coelho et al. [26] compared yields for Soxhlet extraction with that of *n*-hexane and supercritical CO_2_ extractions. SFE was found to produce the higher yields (12.0–12.7%) in comparison with Soxhlet extraction (12.28%). Jokić et al. [47] concluded that grape oil can be completely extracted by SFE at optimal operating conditions (*P* = 400 bar and *T* = 41 °C), resulting with the oil yield of 14.87%. However, here the yield of Soxhlet extraction with *n*-hexane was 14.96%. Bravi et al. [44] used a seed mixture of different red (Merlot, Cabernet Sauvignon, Cabernet Franc and Raboso) and white (Prosecco, Verduzzo, Pinot Grigio, Chardonnay, Pinot Bianco, Bianco) grape varieties in Soxhlet and SC-CO_2_ extraction. The content of the oil that can be extracted with SC-CO_2_ was 14.4% and was slightly lower than with hexane (15.4%). Prado et al. [28] achieved yield of 13.42% using SFE technique at 350 bar, 40 °C and 0.46 kg CO^2^ h^−1^. Da Porto et al. [48] reported Soxhlet extraction using *n*-hexane in a 1:12 ratio for 6 h and UAE in 1:8 ratio at 20 kHz and 50, 100 and 150 W for 30 min against the Soxhlet extraction of grape seeds of Raboso Piave variety. Here, an increase of the ultrasound power from 50 to 150 W caused the yield to jump from 11.42 to 14.08%. Anyhow, Soxhlet extraction had a higher yield of 14.64%, which can be explained by providing freshly condensed solvent for 6 h, while UAE was a batch system that lasted for 30 min. UAE at 150 W for 30 min increased the yield to approximately 14%, which is comparable to the yield of Soxhlet extraction at 70 °C for 6h [48]. In the study conducted by de Menezes et al. [17], oil from Burgundy variety grape seeds was obtained by the Soxhlet technique and UAE technique with hexane. The oil content was 16.28% for the Soxhlet technique and 11.60% for the UAE technique. Böger et al. [22] showed that UAE is useful technique for increasing of the oil yields while reducing the durations of the extractions, while using less solvent and obtaining the high quality oils.

It is important to note that fatty acid composition in grape seed oils may be highly influenced by the grape variety and growing conditions [49]. Since the SFE was selected as the technique with the highest potential for the oil recovery, it was further examined how its operating parameters affect the fatty acid profiling (Table S3). To that end, Prado et al. [28] investigated both lab- and pilot-scale SFE grape samples for Malbec and Cabernet Franc varieties. Although SFE parameters differed among pilot and lab experiments, it was still found that all extracted oils contained the linoleic (71.20%) and oleic acids (15.10%) as the main components. When considering saturated fatty acids, palmitic (8.13%) and stearic (4.05%) acids were determined as the most abundant. Coelho et al. [26] analyzed the fatty acid profiles of the SE/hexane samples and those extracted by SFE by varying different operating conditions. Highest percentages in samples accounted for linoleic (64.5–67.37%), oleic (19.18–20.64%), palmitic (7.38–8.22%) and stearic acids (4.33–5.61%). Judging by our data and the literature, it can be concluded that the fatty acid profile from all samples followed the expected content. However, notable differences in fatty acid profiling were found for seed samples recovered by different extraction techniques. 

The obtained results of functional quality indices highlighted that grape seed oils recovered by SE and UAE expressed the best functional quality. On the other hand, it is important to mention that the aforementioned extracts were obtained using *n*-hexane, which can be evaporated from the oils, but they can still be contaminated by the traces of this organic solvent. In conclusion, SFE stands out as a successful technique for isolation of solvent-free grape seed oil with proper functional quality and without any traces of extraction solvents.

When observing the impact of pressure, it can be concluded that the higher pressure reduced tocopherol content, although higher pressures gave higher extraction yields. The increase in temperature resulted in elevated tocopherol content. While higher temperatures promote higher solubility of the solute and enhance mass transfer of solute from matrix to the SFE solvent, the lowest tocopherol content was found at 50 °C. This can be related to the crossover phenomenon at aforementioned temperature. Bravi et al. [44] have been observed for SFE extracts that an elevated temperature (40 vs. 80 °C) influenced increased α-tocopherol content, due to the higher solubility of α-tocopherol at 80 than at 40 °C. Samples with a reduced particle size (RGS315-SFE) exhibited higher tocopherol content than samples with larger particle size (7.57 vs. 4.60 mg 100 g^−1^). This was expected, as particle size reduction leads to increased extraction efficiency, since the free surface area for mass transfer is increased and diffusional resistance in solid phase is decreased [44]. 

Since the optimal SFE parameters gave the highest overall yield, after adjusting for total tocopherols, results showed that SFE at given conditions was the most successful for exhaustion of grape seeds. In summary, red grape seed oils are richer in α- and γ-tocopherol and higher amount of total tocopherols can be recovered by SFE, but considerable attention must be paid to the proper adjustment of an extraction parameters.

Based on the data, it can be concluded that the antioxidant capacity is strongly dependent upon the applied extraction parameters. Moreover, oils obtained from red grape seeds had a higher antioxidant capacity as compared to those from white grape, probably because higher content of α-tocopherols was also in red grape samples. Tangolar et al. [47] have previously reported higher concentrations of α- and γ-tocopherol in the Cabernet Sauvignon variety than Chardonnay variety. Similar results were noted by Ben Mohamed et al. [9], who evaluated the bioactive compounds and antioxidant activities of six different grape seed oils.

Although several in vitro and in vivo studies have shown that tocotrienol-rich fractions from grape seeds are more potent antioxidants, another study documented that α-tocopherol had higher free radical scavenging activity than tocotrienol-rich fractions, and consisted of a mixture of γ-tocopherol and α- and γ-tocotrienol [12]. This was explained by the purity of tocotrienol-rich fractions, which contains approximately 6% tocols.

Among SFE and SE extracts, the SFE exhibited higher antioxidant capacity for both DPPH and ABTS assays. The opposite findings were reported by Wang et al. [50], who evaluated and compared in vitro antioxidant activities of unsaponifiable fractions of 11 kinds of edible vegetable oils (flaxseed, olive, grape seed, corn, soybean, sunflower seed, walnut, perilla, rapeseed, sesame, and camellia) by DPPH, ABTS and FRAP assays [50]. The authors identified grape seed oils with the lowest total antioxidant capability that might be attributed to the different processing techniques and different amounts of hydrophilic and lipophilic antioxidant content in the samples. Hence, confirming that oil extraction procedure is the crucial element that affect antioxidant activity and overall quality of extracts. A different study by Ben Mohamed et al. [9] found higher ABTS values than our study for red grape oils. Oils were recovered by both SFE and SE with 7.5–8.2 µM and 5.9–6.5 µM Trolox g^−1^, respectively. White grape varieties recovered by SFE had the ABTS values of 4.9–6.0 µM Trolox g^−1^, while SE samples had lower values for antioxidative activity of 4.4–4.9 µM Trolox g^−1^ [9]. In the work of Konuskan et al. [15], the highest DPPH radical scavenging activity was noted for Cabernet Sauvignon variety, which also makes up the highest part in red grape seed mixture used in this work. The authors also found that hydrophilic antioxidant values were unaffected by the extraction method, while lipophilic values were higher for the super critical CO_2_-extracted oils. This suggested that the type of extraction, as well as the corresponding parameters, should be thoroughly considered for the isolation of oil from grape seeds in order to obtain desired antioxidant potential.

## 5. Conclusions

Grape seeds, as a by-product of wine industry, can be successfully valorized as a raw material for recovering oils with high-quality bioactive antioxidants. Modern extraction technologies, such as UAE, MAE and SFE, were compared to Soxhlet extraction for obtaining red and white grape seed oils. The SFE was the best method with respect to extraction yield at optimum processing parameters (350 bar, 60 °C and 0.4 kg h^−1^), providing 12.23% and 11.86% yields for red and white grape seeds, respectively.

A fatty acid profiling of samples identified polyunsaturated fatty acids as dominating in this category of constituents (69.27–74.88%) with linoleic acid (68.61–74.15%) as major representative. Monounsaturated fatty acids were found in lower amounts (13.53–18.62%) where oleic acid was predominant compound (13.39–18.47%). Saturated fatty acids were detected in the lowest amounts ranging from 11.28–12.27%. Different extraction techniques did not alter fatty acid profiles in the samples; however, the application of SFE technology yielded appreciable quantities of tocopherols. The highest antioxidant potential (i.e., DPPH and ABTS) for red and white grape oils were observed for samples recovered by the UAE.

Based on the results, it can be concluded that the application of non-conventional extraction techniques was efficient for recovering of high-quality grape seed oils that were rich in lipid antioxidants. Thus, such extracts could be incorporated into different functional foods, pharmaceuticals or cosmetic products. Non-conventional techniques have environmental benefits, as they stand out as the “green” extractions, due to absence of organic solvents form the process. Hence, preventing numerous disadvantages of conventional alternatives, such as toxic residuals of organic solvents in the extracts, negative environmental impacts and flammability.

## Figures and Tables

**Table 1:** Design of an experiment for grape seeds oil isolation.

Sample	Extraction Technique	Process Conditions
Red grape seeds (RGS)		
RGS-SFE1	Supercritical fluid extraction	250 bar, 40 °C, 0.3 kg h^−1^
RGS-SFE2		300 bar, 40 °C, 0.3 kg h^−1^
RGS-SFE3		350 bar, 40 °C, 0.3 kg h^−1^
RGS-SFE4		350 bar, 50 °C, 0.3 kg h^−1^
RGS-SFE5		350 bar, 60 °C, 0.3 kg h^−1^
RGS-SFE6		350 bar, 60 °C, 0.2 kg h^−1^
RGS-SFE7		350 bar, 60 °C, 0.4 kg h^−1^
RGS-SFE315		350 bar, 60 °C, 0.4 kg h^−1^, 315 < d < 800 µm
RGS-SFE800		350 bar, 60 °C, 0.4 kg h^−1^, d > 800 µm
RGS-UAE	Ultrasound-assisted extraction	solvent: *n*-hexane, 40 kHz, 50 °C, 40 min, 60 W L^−1^
RGS-MAE	Microwave-assisted extraction	solvent: *n*-hexane, 600 W, 15 min
RGS-SE	Soxhlet extraction	solvent: *n*-hexane, 6 h, 15 exchanges of extract
White grape seeds (WGS)		
WGS-SFE	Supercritical fluid extraction	350 bar, 60 °C, 0.4 kg h^−1^
WGS-UAE	Ultrasound-assisted extraction	solvent: *n*-hexane, 40 kHz, 50 °C, 40 min, 60 W L^−1^
WGS-MAE	Microwave-assisted extraction	solvent: *n*-hexane, 600 W, 15 min
WGS-SE	Soxhlet extraction	solvent: *n*-hexane, 6 h, 15 exchanges of extract

**Table 2:** Relative content (%) of fatty acids in red and white grape seed oils obtained by different extraction techniques.

Fatty Acid	Palmitic (C16:0)	Palmitoleic (C16:1)	Stearic (18:0)	Oleic (C18:1n9C)	Linoleic (C18:2n6C)	γ-Linolenic (C18:3n6C)	α-Linolenic (C18:3n3C)	Heneicosanoic (C21:0)	Saturated Fatty Acids	Monounsaturated Fatty Acids	Polyunsaturated Fatty Acids	Unsaturated Fatty Acids	Ratio S/U
Red grape seeds													
RGS-SFE	7.93	0.14	3.79	13.39	73.58	0.18	0.66	0.34	12.06	13.53	74.42	87.94	0.14
RGS-UAE	7.20	0.12	3.83	13.72	74.15	0.21	0.52	0.25	11.28	13.84	74.88	88.72	0.13
RGS-MAE	7.33	0.14	4.33	16.18	71.09	0.24	0.45	0.25	11.91	16.31	71.78	88.09	0.14
RGS-SE	7.42	0.13	3.93	14.03	73.45	0.23	0.55	0.26	11.61	14.16	74.23	88.39	0.13
White grape seeds													
WGS-SFE	7.73	0.14	4.29	17.57	69.37	0.23	0.41	0.25	12.27	17.71	70.02	87.73	0.14
WGS-UAE	7.66	0.17	4.24	18.39	68.65	0.24	0.42	0.23	12.13	18.56	69.31	87.87	0.14
WGS-MAE	7.29	0.13	4.27	17.91	69.83	0.00	0.39	0.19	11.74	18.03	70.22	88.26	0.13
WGS-SE	7.51	0.16	4.37	18.47	68.61	0.25	0.40	0.24	12.11	18.62	69.27	87.89	0.14

**Table 3:** Functional quality indices of grape seed oil obtained by different extraction techniques.

Sample	AI	TI	H/H
Red grape seeds			
SFE7-RGS	0.090	0.257	11.07
SE-RGS	0.081	0.242	12.28
UAE-RGS	0.083	0.258	11.97
MAE-RGS	0.084	0.249	11.86
White grape seeds			
SFE1-WGS	0.088	0.268	11.30
SE-WGS	0.087	0.264	11.42
UAE-WGS	0.083	0.256	12.09
MAE-WGS	0.085	0.264	11.65

AI: atherogenicity index; TI: thrombogenicity index; H/H: ratio between hypocholesterolemic and hypercholesterolemic fatty acids; RGS: red grape seeds; WGS: white grape seeds.

**Table 4:** The influence of different extraction parameters on tocopherol content in red grape seed oils recovered by supercritical fluid extraction at (mg 100 g^−1^).

Sample	Parameter	α-Tocopherol	γ-Tocopherol	Total Tocopherols
	Pressure (bar)			
RGS1-SFE	250	5.65 ± 0.20 ^a^	1.35 ± 0.09 ^a^	7.00 ± 0.29 ^a^
RGS2-SFE	300	5.15 ± 0.10 ^b^	1.29 ± 0.04 ^a^	6.45 ± 0.14 ^b^
RGS3-SFE	350	5.05 ± 0.07 ^b^	1.35 ± 0.02 ^a^	6.40 ± 0.05 ^b^
	Temperature (°C)			
RGS3-SFE	40	5.05 ± 0.07 ^b^	1.35 ± 0.02 ^b^	6.40 ± 0.05 ^b^
RGS4-SFE	50	4.85 ± 0.07 ^b^	1.26 ± 0.02 ^c^	6.11 ± 0.07 ^b^
RGS5-SFE	60	6.18 ± 0.33 ^a^	1.76 ± 0.02 ^a^	7.94 ± 0.31 ^a^
	Solvent flow rate (kg h^−1^)			
RGS6-SFE	0.2	7.84 ± 0.04 ^a^	1.65 ± 0.09 ^a^	9.49 ± 0.09 ^a^
RGS5-SFE	0.3	6.18 ± 0.33 ^b^	1.76 ± 0.02 ^a^	7.94 ± 0.31 ^b^
RGS7-SFE	0.4	5.35 ± 0.10 ^c^	1.16 ± 0.02 ^b^	6.51 ± 0.12 ^c^
	Particle size (µm)			
RGS315-SFE	315–800	6.18 ± 0.33 ^a^	1.39 ± 0.09 ^a^	7.57 ± 0.43 ^a^
RGS800-SFE	>800	3.63 ± 0.03 ^b^	0.98 ± 0.06 ^b^	4.60 ± 0.02 ^b^

* Values with different letters in the same column are significantly different (*p* < 0.05).

**Table 5:** Influence of extraction technique on tocopherols content in grape seed oil (mg 100 g^−1^).

Sample	α-Tocopherol	γ-Tocopherol	Total Tocopherols
Red grape seeds			
RGS-SFE	5.35 ± 0.10 ^b^	1.16 ± 0.02 ^b^	6.51 ± 0.12 ^b^
RGS-SOX	4.85 ± 0.06 ^c^	1.48 ± 0.06 ^a^	6.33 ± 0.03 ^c^
RGS-UAE	6.51 ± 0.05 ^a^	1.41 ± 0.04 ^a^	7.92 ± 0.04 ^a^
RGS-MAE	6.51 ± 0.09 ^a^	1.44 ± 0.04 ^a^	7.96 ± 0.04 ^a^
White grape seeds			
WGS-SFE1	0.44 ± 0.03 ^c^	0.51 ± 0.04 ^c^	0.95 ± 0.07 ^d^
WGS-SOX	1.47 ± 0.13 ^b^	0.90 ± 0.09 ^a^	2.37 ± 0.04 ^b^
WGS-UAE	1.47 ± 0.03 ^b^	0.71 ± 0.02 ^b^	2.18 ± 0.02 ^c^
WGS-MAE	1.90 ± 0.03 ^a^	0.73 ± 0.04 ^b^	2.63 ± 0.02 ^a^

* Values with different letters in the same column are significantly different (*p* ≤ 0.05).

**Table 6:** Influence of SFE parameters on antioxidant activity of red grape seed oil (µM Trolox g^−1^).

Sample	Parameter	DPPH	ABTS
	Pressure (bar)		
RGS1-SFE	250	1.46 ± 0.36 ^a^	6.26 ± 0.17 ^a^
RGS2-SFE	300	1.35 ± 0.07 ^a^	3.14 ± 0.18 ^b^
RGS3-SFE	350	1.58 ± 0.16 ^a^	3.75 ± 0.56 ^b^
	Temperature (°C)		
RGS3-SFE	40	1.58 ± 0.16 ^a^	3.75 ± 0.56 ^a^
RGS4-SFE	50	1.61 ± 0.12 ^a^	3.66 ± 0.10 ^a^
RGS5-SFE	60	1.43 ± 0.18 ^a^	3.78 ± 0.32 ^a^
	Solvent flow rate (kg CO_2_ h^−1^)		
RGS6-SFE	0.2	1.87 ± 0.04 ^a^	4.12 ± 0.40 ^a,b^
RGS5-SFE	0.3	1.43 ± 0.18 ^b^	3.78 ± 0.32 ^b^
RGS7-SFE	0.4	2.25 ± 0.24 ^a^	4.92 ± 0.33 ^a^
	Particle size (µm)		
RGS315-SFE	315–800	1.75 ± 0.55 ^a^	4.60 ± 0.20 ^a^
RGS800-SFE	>800	1.36 ± 0.25 ^a^	3.74 ± 0.11 ^b^

* Values with different letters in the same column are significantly different (*p* ≤ 0.05). DPPH: scavenging activity towards DPPH radicals; ABTS: scavenging activity towards ABTS^+^ radicals.

## Supplementary Materials

The following are available online at https://www.mdpi.com/2076-3921/9/7/568/s1, Table S1: Influence of different extraction parameters on tocopherol yield in red grape seed oil samples (mg 100 g^−1^); Table S2: Influence of different extraction techniques on tocopherol yield in grape seed oil (mg 100 g^−1^); Table S3: Relative content of fatty acids (%) in all obtained samples.
