# Supplementary material for: Innovative and Conventional Valorizations of Grape Seeds from Winery By-Products as Sustainable Source of Lipophilic Antioxidants
Source: Antioxidants (Basel). 2020 Jul 1;9(7):568. doi: 10.3390/antiox9070568 (PMC7402152; doi:10.3390/antiox9070568)
Supplement: Supplementary file 1 [file antioxidants-09-00568-s001.pdf]

Table S1. Influence of different extraction parameters on tocopherol yield in red grape seed oil samples (mg 100 g^{-1})

Sample	Parameter	α-tocopherol	γ-tocopherol	Total tocopherols
	Pressure [bar]			
RGS1-SFE	250	0.421	0.101	0.522
RGS2-SFE	300	0.428	0.107	0.536
RGS3-SFE	350	0.441	0.118	0.559
	Temperature [°C]			
RGS3-SFE	40	0.441	0.118	0.559
RGS4-SFE	50	0.418	0.109	0.526
RGS5-SFE	60	0.605	0.172	0.778
		Solvent flow rate [kg/h]		
RGS6-SFE	0.2	0.617	0.130	0.747
RGS5-SFE	0.3	0.605	0.172	0.778
RGS7-SFE	0.4	0.654	0.142	0.796
	Particle size [µm]			
RGS315-SFE	315-800	0.340	0.076	0.416
RGS800-SFE	>800	0.384	0.104	0.487

Table S2. Influence of different extraction techniques on tocopherol yield in grape seed oil (mg 100 g^{-1})

Sample	α-Tocopherol	γ-Tocopherol	Total tocopherols
**Red grape seeds**			
RGS-SFE	0.654	0.142	0.796
RGS-SOX	0.447	0.136	0.583
RGS-UAE	0.618	0.134	0.752
RGS-MAE	0.636	0.141	0.778
**White grape seeds**			
WGS-SFE1	0.052	0.060	0.113
WGS-SOX	0.132	0.081	0.212
WGS-UAE	0.122	0.059	0.181
WGS-MAE	0.207	0.079	0.286

Table S3. Relative content of fatty acids (%) in all obtained samples

**Fatty acid**	**Palmitic (C16:0)**	**Palmitooleic (C16:1)**	**Stearic (18:0)**	**Oleic (C18:1n9C)**	**Linoleic (C18:2n6C)**	**γ-Linolenic (C18:3n6C)**	**α-Linolenic (C18:3n3C)**	**Heneicosanoic (C21:0)**	**Saturated fatty acids**	**Monounsaturated fatty acids**	**Polyunsaturated fatty acids**	**Unsaturated fatty acids**	**Ratio S/U**
**Red grape seeds**													
RGS-SFE1	8.10	0.15	3.84	13.54	73.48	0.22	0.66	0.00	11.94	13.69	74.36	88.06	0.14
RGS-SFE2	7.76	0.15	3.64	13.42	74.40	0.00	0.63	0.00	11.40	13.57	75.03	88.60	0.13
RGS-SFE3	7.64	0.14	3.79	13.57	74.00	0.00	0.60	0.27	11.69	13.71	74.60	88.31	0.13
RGS-SFE4	7.94	0.14	3.85	13.61	73.35	0.21	0.61	0.29	12.08	13.75	74.17	87.92	0.14
RGS-SFE5	8.04	0.15	3.78	13.94	72.82	0.22	0.75	0.31	12.12	14.09	73.79	87.88	0.14
RGS-SFE6	7.73	0.14	3.74	13.68	73.61	0.20	0.61	0.27	11.75	13.82	74.43	88.25	0.13
RGS-SFE7	7.93	0.14	3.79	13.39	73.58	0.18	0.66	0.34	12.06	13.53	74.42	87.94	0.14
RGS-SFE315	7.98	0.16	3.65	13.45	73.48	0.21	0.72	0.34	11.98	13.61	74.41	88.02	0.14
RGS-SFE800	7.53	0.12	3.95	13.74	73.83	0.00	0.57	0.27	11.74	13.86	74.40	88.26	0.13
RGS-UAE	7.20	0.12	3.83	13.72	74.15	0.21	0.52	0.25	11.28	13.84	74.88	88.72	0.13
RGS-MAE	7.33	0.14	4.33	16.18	71.09	0.24	0.45	0.25	11.91	16.31	71.78	88.09	0.14
RGS-SOX	7.42	0.13	3.93	14.03	73.45	0.23	0.55	0.26	11.61	14.16	74.23	88.39	0.13
**White grape seeds**													
WGS-SFE	7.73	0.14	4.29	17.57	69.37	0.23	0.41	0.25	12.27	17.71	70.02	87.73	0.14

WGS-UAE	7.66	0.17	4.24	18.39	68.65	0.24	0.42	0.23	12.13	18.56	69.31	87.87	0.14
WGS-MAE	7.29	0.13	4.27	17.91	69.83	0.00	0.39	0.19	11.74	18.03	70.22	88.26	0.13
WGS-SOX	7.51	0.16	4.37	18.47	68.61	0.25	0.40	0.24	12.11	18.62	69.27	87.89	0.14
